# The Effect of Naturalistic Developmental Behavioral Interventions and Aided AAC on the Language Development of Children on the Autism Spectrum with Minimal Speech: A Systematic Review and Meta-analysis

**DOI:** 10.1007/s10803-024-06382-7

**Published:** 2024-06-07

**Authors:** Lauramarie Pope, Janice Light, Emily Laubscher

**Affiliations:** 1 Department of Speech, Language, and Hearing Sciences, Auburn University, 1199 Haley Center, Auburn, AL 36849 USA; 2 Department of Communication Sciences and Disorders, The Pennsylvania State University, University Park, USA; 3 Department of Speech-Language Pathology, Regis College, Weston, USA

**Keywords:** Autism spectrum disorder, Naturalistic developmental behavioral interventions, Augmentative and alternative communication, Language development, Systematic review

## Abstract

Both naturalistic developmental behavioral interventions (NDBIs) and augmentative and alternative communication (AAC) have been shown to support the language development of children with a diagnosis of autism spectrum disorder and minimal speech. However, little research has addressed the impact of incorporating AAC systems within NDBIs. This systematic review was conducted to assess the relative impact of NDBI procedures *with* and *without* AAC on the language development of children on the autism spectrum with minimal speech. Relevant studies were located through systematic database searching, targeted review of relevant journals, and ancestral search of references from identified and associated papers. Relevant study characteristics were coded for all included studies, as well as determining certainty of evidence and calculating effect sizes for language variables. All procedures followed the systematic review guidelines set by the Cochrane Collaboration. A total of 29 relevant studies were included within this review, covering both single-case and group design research. Three studies were identified that directly compared NDBI and AAC interventions. NDBIs had a strong impact on language across study types (i.e., with and without AAC), though both aggregate and comparative effect sizes were notably larger when AAC was included within NDBI procedures, as compared to NDBIs without AAC. Results suggest that combining AAC with NDBI procedures may lead to better language outcomes than NDBIs alone for children on the autism spectrum with minimal speech.

Children on the autism spectrum often experience significant language and communication challenges (Tager-Flusberg et al., 2005). In fact, over 40% of children with a diagnosis of autism spectrum disorder (ASD) do not develop speech that is functional to meet their daily communication needs (Centers for Disease Control and Prevention [CDC], 2007). Naturalistic developmental behavioral interventions (NDBIs) have demonstrated positive impacts on development across a variety of domains (e.g., social engagement, play, cognition) for children on the autism spectrum, including language specifically (Crank et al., 2021; Sandbank et al., 2020; Tiede & Walton, 2019).

NDBIs blend developmental principles with behavioral strategies and include three core features: (a) the type and progression of targeted skills, (b) the contexts of intervention, and (c) the strategies used to promote growth across learning domains (Schreibman et al., 2015). NDBIs are designed to target skills across developmental domains (e.g., language, social, cognition, motor, play), as opposed to focusing on a single skill—or domain—in isolation (Landa et al., 2011). NDBIs also focus on a developmental sequence of skill acquisition (Schreibman et al., 2015), targeting skills in one domain (e.g., joint engagement) that build and intersect with other skills within and across domains (e.g., attention, expressive language) to support an integrated developmental cascade. NDBIs are designed to be situated within the context of a child’s natural environment and routines (Schreibman et al., 2015), aligning with the integrated nature of intervention goals. The focus of NDBIs is to promote high levels of success within interactive contexts, increasing the child’s participation and independence over time. Thus, NDBIs use a range of behavioral strategies across target domains (e.g., modeling, shaping, prompting, expanding, reinforcement) to support a child’s success in completing functional, integrated skills within naturalistic routines (Schreibman et al., 2015).

Many established interventions fall under the umbrella category of an “NDBI,” including Incidental Teaching (IT; Hart & Risley, 1968), Pivotal Response Training (PRT; Koegel & Koegel, 2006), the Early Start Denver Model (ESDM; Dawson et al., 2010), Enhanced Milieu Teaching (EMT; Kaiser & Hester, 1994), Project ImPACT (Improving Parents As Communication Teachers; Ingersoll & Wainer, 2013), Joint Attention Symbolic Play Engagement and Regulation (JASPER; Kasari et al., 2006), and Early Achievements (EA; Landa et al., 2011), though this list is not exhaustive (Schreibman et al., 2015). However, intervention procedures that do not officially fall under these specific manualized protocols may still qualify as NDBIs, given that they: (a) occur in a natural context; (b) employ natural contingencies; (c) include integration of developmental principles; and (d) include integration of behavioral principles (Tiede & Walton, 2019).

NDBIs appear to contribute to a measurable increase in language growth for many young children on the autism spectrum. However, children on the spectrum with minimal speech are less likely to benefit from NDBIs in their traditional format than children on the spectrum with functional speech (Kasari et al., 2014). Without speech, these children have limited means to engage in the rich language learning environment of NDBIs. In fact, one study found that even with relatively intensive intervention (2 h per week over the course of 12 weeks), 40% of children with limited speech made minimal progress when exposed to established NDBI strategies (Kasari et al., 2014).

Without an effective means to communicate, these children are at risk in all aspects of development, including education, family and peer interactions, and community participation (Light & McNaughton, 2012). The available evidence indicates that aided augmentative and alternative communication (AAC) systems are highly effective at supporting the communication of children on the autism spectrum with minimal speech (Ganz et al., 2012; Iacono et al., 2016). Aided AAC refers to any means of communication other than speech that relies on some type of external support to assist communication (e.g., letter boards, a speech generating app on a tablet, picture exchange).

However, most manualized NDBI interventions target spoken language (or possibly gestures and vocalizations) almost exclusive, and do not explicitly incorporate aided AAC systems or supports into their procedures (see Kasari et al., 2014 for a notable exception). Importantly, evidence suggests that providing AAC may actually bolster speech outcomes (e.g., Kasari et al., 2014; Romski et al., 2010; Schlosser & Wendt, 2008). Several studies have also offered concrete guidance for integrating aided AAC into naturalistic interventions for children on the autism spectrum specifically (Ganz et al., 2019), as well as children with developmental disabilities and minimal speech more generally (Kaiser & Wright, 2013).

A robust evidence base demonstrates that NDBIs benefit the language development of children on the autism spectrum (Crank et al., 2021; Sandbank et al., 2020; Tiede & Walton, 2019). There is also evidence that aided AAC intervention positively impacts both the speech and language development of children on the autism spectrum with minimal speech (Ganz et al., 2012; Iacono et al., 2016). However, to date, no study has comprehensively addressed the extant research in these two domains to investigate the relative contributions of NDBIs and AAC on the language development of young children with a diagnosis of ASD. The goal of the present systematic review was to bridge the research on NDBIs and that on aided AAC interventions by systematically investigating the relative impact of NDBI procedures and aided AAC on language growth for children on the autism spectrum. The following questions were addressed within this review: 1) What is the effectiveness of NDBIs *without* aided AAC as a component (NDBI-Only) on the language development of children on the autism spectrum?; 2) What is the effectiveness of aided AAC interventions that qualify as NDBIs (NDBI + AAC; per criteria set by Tiede & Walton, 2019) on the language development of children on the autism spectrum?; and 3) What is the comparative effectiveness of NDBI-Only and NDBI + AAC interventions?

## Methods

Systematic reviews are an effective tool for synthesizing an existing evidence base to draw broader conclusions about a specific research question, as well as guide future research (Snyder, 2019). All procedures followed the systematic review guidelines set by the Cochrane Collaboration (http://www.cochrane.org). The review protocol and codebook are available upon request.

### Inclusion Criteria

Inclusion criteria for included studies comprised the following: (a) published in English; (b) peer-reviewed; (c) all participants had a diagnosis of ASD or had characteristics of ASD (or data for participants on the autism spectrum could be extracted) and no additional concomitant developmental disability diagnoses (e.g., cerebral palsy, Down syndrome); (d) participants were 13 years old or younger; (e) investigated an intervention; (f) met NDBI criteria as set by Tiede and Walton (2019): natural contexts, natural contingencies, integration of developmental principles, integration of behavioral principles; (g) language measures were included as a dependent variable(s); (h) research design was a single-case or group design with experimental control (single case: baseline and at least three demonstrations of effect measured over time; group: equivalent experimental and comparison groups); (h) sufficient data were included for calculating effect sizes; and (h) education/research professionals served as direct interventionists with participants (i.e., caregiver-mediated or peer-mediated interventions were excluded).

Participants with concomitant additional developmental disabilities (e.g., cerebral palsy, Down syndrome) were excluded in order to focus specifically on the impact of NDBIs on children with a primary diagnosis of ASD across all study types. Participants above age 13 were excluded as NDBIs are typically targeted at younger children. However, no studies were excluded based upon these criteria, though a small number of individual participants from included single-case studies were excluded from this review based upon these criteria. The review was limited to professional interventionists (e.g., researchers, teachers, teaching assistants, clinical therapists). Focusing on professional interventionist only and excluding caregiver- and peer-mediated interventions allowed for better comparison across different intervention types, as it controlled for factors related to the interventionist (e.g., formal training working with children on the autism spectrum). Additionally, some evidence suggests that caregiver-mediated NDBIs may not be as effective on language development as those completed by education professionals (Sandbank et al., 2020). However, studies that included additional caregiver training were included if caregivers did not serve as the main interventionist. Studies that included teacher training/coaching in NDBI procedures were included provided that there was evidence that the teachers implemented the procedures with fidelity (i.e., 80% or greater) during intervention. All publication dates within the targeted electronic databases were included. Both single-case and group design studies were included, as NDBI research tends to use more group design methods, and AAC literature is predominantly single-case. Thus, in order to cover both areas sufficiently, all relevant experimental research was considered. Single case and group studies were analyzed separately.

### Search Procedures

**Fig. 1:**
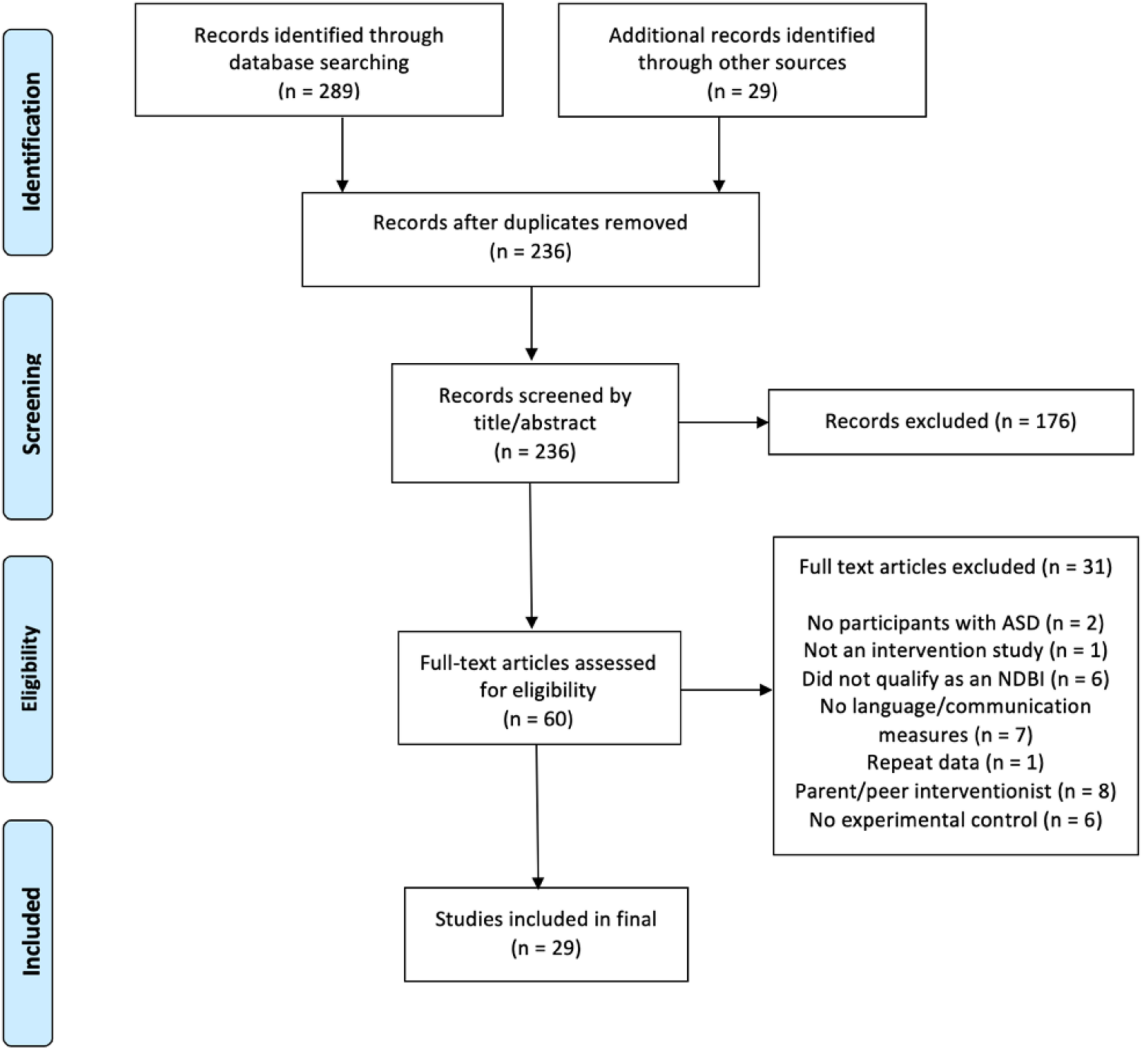
Search strategy

Relevant studies were located through systematic database searching, targeted review of relevant journals, and ancestral search of references from identified and associated papers. The following databases were consulted: ERIC, PsychInfo, PubMed, and Taylor & Francis (which contains all issues of *Augmentative and Alternative Communication*). The *Journal of Applied Behavior Analysis* and all journals published by the American Speech-Language-Hearing Association were also systematically searched, as they are frequent publication locations for both NDBI and AAC studies but are not fully covered in the targeted database searches (see Fig. 1 for an illustration of the search procedures). The original search was conducted in July 2021 and updated in December 2022.

The following search terms were used for all database and journal searchers: (a) autism OR autistic OR ASD AND (b) NDBI OR naturalistic OR “project impact” OR PRT OR “pivotal response” OR “incidental teaching” OR ESDM OR “early start Denver model” OR EMT OR “enhanced milieu” OR JASPER OR “early achievements” AND (c) language OR AAC OR augmentative OR augmented OR SGD. These procedures identified 289 total references across all databases and journals. An additional 29 potential articles were located through ancestral review and associated papers (e.g., previous published NDBI meta-analyses). After duplicates were removed, 236 unique articles were screened by title and abstract. Application of specific inclusion criteria led to the exclusion of 176 articles. Full text review of the remaining 60 papers resulted in 29 studies that met the inclusion criteria and were included in this review.

### Data Extraction and Coding

Coding procedures were adapted from Schlosser and Wendt (2008), with consideration to the relevant characteristics of NDBI studies, as well as single-case vs. group research design. Data extracted from all studies included: (a) Study Identification (i.e., first author name, date of publication, and design type); (b) Participant Characteristics (i.e., ID, age, gender, baseline language measures); (c) Intervention Characteristics (i.e., type of NDBI, interventionist, context/location of intervention, AAC system); (d) Dependent Variable Characteristics (only including language/communication measures—expression or comprehension, language domain: social communication, requesting, semantic, syntactic); (e) Outcome Measure(s) (i.e., gain scores, Tau-U, Cohen’s *d*); and (f) Certainty of Evidence.

Outcomes for single-case research were calculated at the individual participant level and included two measures of effect size: gain scores and Tau-U. Gain scores were included as they offer a straightforward visual representation of average change from baseline to intervention and are a common effect size index in both the NDBI and AAC literature. Gain scores were calculated by participant for each measure by taking the difference between the average of baseline and the average of intervention for included outcome measures. Tau-U was also calculated, as it captures both potential baseline trends as well as overlap of values across phases (Parker et al., 2011), making it a more sensitive measure in assessing change from baseline to intervention than other single-case effect size measures that only take phase overlap into account. Additionally, Tau-U is a common single-case effect size in both AAC and NDBI research. Thus, the use of this single-case effect size may make the results more accessible and interpretable across both fields. Tau-U values comparing baseline and intervention for each included outcome measure by participant were calculated using a free online calculator (Vannest et al., 2011). Tau-U values below 0.2 are considered a small effect, values of 0.21–0.59 represent a medium effect, 0.6–0.79 a large effect, and 0.8 or greater a very large effect (Vannest & Ninci, 2015).

Group study outcomes were calculated using Cohen’s *d* (Cohen, 1992). Comparison values represented either magnitude of change from pre to post intervention for the experimental vs. control group, or comparison of post-intervention values between the two groups, depending upon the reported data. Effect size values of 0.2 are considered a small difference, 0.5 a medium difference, and 0.8 a large difference between groups (Cohen, 1992). For studies with unequal sample sizes, Hedge’s g was also calculated, but results were indistinguishable from Cohen’s *d* values.

Certainty of evidence was assessed following guidelines from Reichow et al. (2008). These procedures were developed specifically for experimental research in autism intervention and include standards for both single-case and group designs (Reichow et al., 2008), making these procedures directly applicable to the goals of the current study. Various study design, implementation, analysis, and reporting quality indicators were assessed, including: (1) description of participants, settings, and the dependent variable(s); (2) demonstration of experimental control; (3) interrater reliability of the data and procedural fidelity of the intervention; and (4) generalization, maintenance, and social validity of the procedures and results. Based upon these indicators, evidence was rated as strong, adequate, or weak (see Reichow et al., 2008 for an overview of the process).

### Meta-analysis

Effect sizes were calculated for every language outcome measure in each included study. Tau-U was calculated at the individual participant level for single-case designs (e.g., comparing baseline measures of included language outcomes to intervention measures for each participant) and Cohen’s *d* was calculated at the group level for group designs (e.g., comparing outcomes for the experimental and control groups on included language measure(s)). Study-level effect size values were calculated for studies with multiple included outcome measures by taking the weighted average of all included effect size values in that study, weighted by the number of paired comparisons in each included outcome measure within that study (Parker et al., 2011). Weighted averages of single-case studies were calculated using a free online calculator (Vannest et al., 2011). For group studies, weighted averages were calculated using R statistical software (R Core Team, 2023).

Effect size values from individual studies were then aggregated within each study design (i.e., single-case or group) by intervention type (NDBI-Only, NDBI+AAC) to provide an average effect size for each design x intervention category. Average effect size values were calculated for 1) single-case NDBI+AAC studies, 2) single-case NDBI-Only studies, and 3) group NDBI-Only studies. Random effects models in R (R Core Team, 2023) were used to estimate average effect sizes across the three design x intervention categories. Given the inherent variability across included studies, random (vs. fixed) effects models were considered appropriate (Del Re, 2015). Two random effects models were run for each design x intervention category—one model including all relevant studies, and one model including only studies rated as having “strong” certainty of evidence.

Three group design studies identified through the search procedures represented direct comparisons of an NDBI without AAC to an AAC intervention (Comparative studies). The reported Cohen’s *d* effect size values for these three studies reflect the outcomes of the AAC intervention group on the included language outcomes as compared to the NDBI group. Given the differences in the specific nature of the comparisons targeted by the three Comparative studies, an average effect size was not calculated for the Comparative studies.

Average effect sizes were used to evaluate: 1) the effectiveness of NDBI-Only interventions; 2) the effectiveness of NDBI+AAC interventions; and 3) the comparative effectiveness of NDBI-Only and NDBI+AAC interventions, including comparison of the average effect size values from NDBI-Only and NDBI+AAC studies, as well as the effect sizes of Comparative studies. Evaluation was based on general criteria set by Cohen (1992) and Vannest and Ninci (2015).

Additionally, the degree to which the inclusion of aided AAC moderated outcomes of NDBIs on language outcomes was investigated using meta-analytic methods, including all single-case studies (NDBI+AAC and NDBI-Only). Moderator analysis was completed via meta-regression in R (R Core Team, 2023), using a mixed effects model.

Lastly, funnel plots were created using R (R Core Team, 2023) to assess potential publication bias of included studies. Separate funnel plots were constructed for NDBI+AAC single-case studies, NDBI-Only single-case studies, and NDBI-Only group studies.

### Data Reliability

All search procedures, data extraction and variable coding, and effect size calculations were completed by the first author. To ensure reliability of the data, the third author completed inclusion criteria assessments (10% of studies), data extraction and variable coding (15% of studies), and certainty of evidence assessments (7% of studies) for a subset of included studies. Reliability calculations were completed by taking the number of agreements divided by the number of agreements plus disagreements, multiplied by 100. There was 100% agreement for adherence to inclusion criteria. Overall agreement for data extraction and variable coding was 94%. Agreement on certainty of evidence assessments was 100%. Disagreements for data extraction and variable coding were discussed and clarified, though original coding values were maintained in all cases.

## Results

A total of 29 studies met inclusion criteria and were included in this review, consisting of 17 single-case and 12 group design studies (see Table 1 for single-case results and Table 2 for group design results). In total, all studies represented 517 total participants on the autism spectrum (54 single-case, 463 group). Three single-case studies included participants with disabilities other than—or in addition to—a diagnosis of ASD (e.g., Klinefelter syndrome, Down syndrome). These participants were excluded from analyses but did contribute to quality assessment related to experimental control (e.g., three demonstrations of an intervention effect).

**Table 1:** Summary of coded variables for included single-case studies

Study authors; design	Participants		Intervention characteristics			DV characteristics	Outcomes		
	ID: age, gender	Language description	NDBI	Partner: context, location	AAC system	Expression or comprehension, language domain: DV	Gain score	Tau U	Certainty of evidence
*NDBIs Without AAC (NDBI-Only)*									
D’Agostino et al. (2020); MPP	Aaron: 4;1, M	One-word requests	IT	Teacher: play, classroom	N/A	Expression, syntactic: independent 3-word requests	+ 10.2	1	Strong
Hampton et al. (2019); MBP	Musa: 7;2, M Tahir: 5;11, M Imram: 7;10, M	< 10 spoken words	EMT	Researcher: play, playroom at school	N/A	Expression, semantic: # different words Expression, social communication: # spontaneous utterances	+ 4.4 + 11.9 + 6.5 + 6.4 + 7.7 + 5.5	0.58 0.99 0.82 0.63 0.95 0.76	Strong
Hampton et al., (2021); MBP	Ken: 4;9, M Jack: 4;9, M Sean: 3;5, M	< 25 words in 20 min	JASP + EMT	Researchers: play, clinic	N/A	Expression, social communication: # total social language utterances Expression, social communication: # spontaneous social language utterances	+ 27.6 + 21.6 + 16.8 + 14.9 + 19.3 + 10.4	0.91 0.77 0.81 0.72 0.81 0.69	Strong
Hancock and Kaiser (2002); MBP	A: 4;2, M B: 3;2, F C: 2;11, M D: 4;6, M	Language age 20–28 months (SICD)	EMT	Researchers: play, clinic	N/A	Expression, syntactic: # total use of language targets (individualized by participant: 2–3 word phrases)	+ 8.8 + 19 + 16.9 + 6.3	0.68 1 1 1	Strong
Harjusola-Webb and Robbins (2012); MBP	Ian: 3;4, M Dennis: 2:11, M Max: 2;9, M	Language age 8–16 months (Vineland)	PRT/EMT	Teachers: various routine activities, classroom	N/A	Expression, social communication: # gestures, vocalizations, words (composite)	+ 61.6 + 47.3 + 67.9	0.93 1 0.94	Adequate • No procedural reliability • No maintenance or generalization
Ingersoll and Schreibman (2006); MBP	Connor: 3;5, M Lena: 3;9, F Heather: 2;5, F Nathan: 2;10, M Jason: 2;10, M	Language age < 8–25 months (MCDI)	PRT	Researchers: play, clinic	N/A	Expression, social communication: # spontaneous language utterances	− 3.8 + 70.8 + 0.8 + 2.7 + 18	− 0.23 0.99 0.12 0.23 0.57	Strong
Ingersoll et al. (2017); MBP	Child 1: 3, M Child 2: 5;4, M Child 3: 2;8, M Child 4: 2;11, M Child 5: 5;2, M Child 6: 3;8, F Child 7: 4;4, F Child 8: 7;9, M Child 9: 6;11, M	Language age 13–37 months (PLS)	ImPACT	Researchers: play, clinic	N/A	Expression, social communication: utterances/min (individualized by participant: word approximations to sentences)	+ 0.7 + 1.4 + 0.6 + 0.6 + 1.1 + 2.3 + 0.4 + 1.2 + 0.9	0.92 1 0.81 0.84 0.97 0.86 0.54 1 0.73	Adequate • Less than 3 baseline measurements for 2 participants • Slightly rising baseline for 2 participants
Kim et al. (2020); MP across stimuli	Jo: 3, M Sol: 6, M	2–3 word phrases	EMT	Researcher: play •Bubbles, truck, playdough •Playdough, bubbles, train Home	N/A	Expression, social communication: # unprompted target words	+ 3.6 + 3.7 + 3.3 + 4.1 + 4.3 + 4.2	1 1 1 1 1 1	Strong
Lane et al. (2016); MPP	Roberto: 5, M	Language age of 12–17 months (HELP)	Naturalistic language intervention	Teacher: play, classroom	N/A	Expression, request: # target requests Expression, request: # novel requests Expression, request: # expanded requests Expression, request: # spontaneous requests Expression, social communication: # spontaneous comments	+ 4.5 + 2.6 + 0.5 + 0.6 + 0	0.73 0.73 0.36 0.36 − 0.05	Strong
Lane et al. (2020); MPP	Samuel: 8, M Conner: 6, M	Minimal expressive language	Naturalistic language intervention	Researcher: play, classroom	N/A	Expression, request: % correct independent expanded requests	+ 43% + 14%	0.58 0.45	Strong
*NDBIs Including AAC (NDBI + AAC)*									
Alzrayer et al. (2021); MBP	Erin: 5, M Joseph: 6, M Mary: 4, F	< 10 spoken words	Natural language paradigm (differential reinforce for vocal vs. SGD)	Teachers: play, classroom	GoTalk Now with 6–9 grid of preferred toys (Symbolstix)	Expression, request: spontaneous vocalizations to request/min Expression, request: spontaneous SGD activations to request/min	+ 1.2 + 1.3 + 1.3 + 0.5 + 0.3 + 0.2	0.98 1 1 0.77 0.5 0.53	Strong
Carnett et al. (2020); MBP	Ryan: 10, M Seth: 5, M	Minimal speech	Teaching “where” questions within natural contexts	Researcher: • Board game, school conference room • Making lemonade, Clinic	• Proloquo2Go with Symbolstix arranged in folders • Proloquo2Go keyboard	Expression, social communication: type “where” sentence Expression, social communication: select “where” + symbol	+ 0.6 + 0.3	0.59 0.33	Strong
Drager et al. (2006); MB across symbol sets + activities	Maggie: 4;5, F Sam: 4;0, M	< 10 spoken words	Aided language modeling	Researcher and SLP: play • Dollhouse, school playset, playground • Cars, dollhouse, playground Daycare	4 PCS, arranged on a board in a square (12 symbols total per participant)	Comprehension, semantic: # correct symbol comprehension Expression, semantic: # correct symbol production	+ 4.4 + 3 + 3.8 + 3 + 7 + 4.8 + 1.6 + 1.8 + 1.8 + 1.5 + 7.7 + 5	0.9 0.77 0.68 0.58 1 1 0.6 0.75 0.44 0.46 1 1	Adequate • Baseline measurements missing right before transition to intervention for two activities for one participant
Finke et al. (2017); MPP	Corey: 12, M Tiffany: 10, F Zach: 9, M Pamela: 13, F Mark: 10, M Evan: 12, M	“First words” stage	Least-to-most prompting	Researcher: storybook reading, classroom	Proloquo2Go: topic page for each book (8) of 25–30 digital images, organized semantic-syntactically	Expression, syntactic: # multi-symbol messages (related to storybook, any modality)	+ 9.1 + 5.2 + 8.7 + 14.4 + 8.4 + 7.4	0.89 0.92 1 1 0.75 0.97	Adequate • Unclear if any differences existed between books chosen during baseline and intervention
Johnston et al. (2003); MPP	Brad: 4;3, M	Language age of 22 months (E-LAP)	Naturalistic intervention strategy	Researcher: play, classroom	1 PCS for “Can I play?” on a 5 × 5 laminated paper key	Expression, social communication: % correct unprompted use of graphic symbol or speech to request entrance to play activity	+ 43%	0.72	Strong
Olive et al. (2007); MPP	Mickey: 3;9, M Terrence: 5;6, M Rocky: 4, M	Minimal speech	EMT	Teacher or teaching assistant: play, classroom	CheapTalk 4 button VOCA with prerecorded requests, and corresponding “pictures” on each button	Expression, request: # independent VOCA requests Expression, request: # total independent requests (VOCA, gesture, or vocalization)	+ 9.2 + 6.2 + 12.8 + 11.9 + 27.8 + 8.9	0.88 0.92 1 1 0.92 0.6	Strong
Schepis et al. (1998); MPP and activities	Ben: 5, M Cory: 5, M Lynn: 3, F Ian: 3, M	Minimal speech	Naturalistic teaching + introduction of VOCA	Teacher and teaching assistants: • Snack • Play Classroom	CheapTalk VOCA with 4–8 buttons, black and white line drawings + text, and prerecorded messages (requests, y/n, thank you)	Expression, social communication: communicative interactions/min (VOCA activation, gestures, vocalizations)	+ 2.8 + 2.4 + 3.7 + 3.1 + 3.4 + 2.5	1 1 1 1 1 1	Adequate • Rising final baseline points for two participants • Low scores on IOA and procedural fidelity • No generalization/ maintenance

*NDBI* naturalistic developmental behavioral intervention, *AAC* augmentative and alternative communication, *DV* dependent variable (language only), *MPP* multiple probe across participants, *IT* Incidental Teaching, *MBP* multiple baseline across participants; *EMT* Enhanced Milieu Teaching, *JASP* Joint Attention, Symbolic Play, *SCID* Sequenced Inventory of Communication Development, *PRT* Pivotal Response Training, *MCDI* MacArthur-Bates Communicative Development Inventory, *PLS* Preschool Language Scales, *ImPact* Improving Parents as Communication Teachers, *HELP* Hawaii Early Learning Profile, *SGD* speech generating device, *SLP* speech-language pathologist, *PCS* picture communication symbols, *E-LAP* Early Learning Accomplishment Profile, *VOCA* voice output communication aid, *IOA* interobserver agreement

**Table 2:** Summary of coded variables for included group design studies

Study authors; design	Participants				Intervention characteristics			DV characteristics	Outcomes^1^ Cohen’s *d*	Certainty of evidence
	N	Age in months: M (SD)	% Female	Language description	NDBI	Partner: context, location	AAC system	Expression or comprehension, language domain: DV		
*NDBIs Without AAC (NDBI-Only)*										
Chang et al. (2016); RCT	35 28	Ex: 48.9 (6.3) Con: 51.6 (6.5)	21 12	Mean language age ~ 30 months (MSEL)	JASPER	Teachers and teaching assistants: play, classroom	N/A	Expression, social communication: # spontaneous language IJA 1-word 2-word 3-word + Expression, request: # spontaneous requests 1-word 2-word 3-word +	0.62 0.66 0.48 0.64 0.2 0.16	Strong
Dawson et al. (2010); RCT	24 24	Ex: 23.9 (4) Con: 23.1 (3.9)	29% (total)	Mean t-scores 21–26 months (MSEL – test mean 50, SD 10)	ESDM + caregiver training	Researchers + caregivers: various activities, clinic and home	N/A	Expression, composite: MSEL expressive Comprehension, composite: MSEL receptive	0.24 0.58	Strong
Engelstad et al. (2020); RCT	15 16	Ex: 48 (8) Con: 46 (6.7)	40 19	Mean language age 20–30 months (MSEL)	EA	Teachers: shared book reading, classroom	N/A	Expression/comprehension,composite: MSEL verbal composite Expression, social communication: frequency of spontaneous vocalizations	0.5 0.74	Strong
Gengoux et al. (2019); RCT	23 20	Ex: 49.5 (11.2) Con: 47.2 (10)	10 18	Significant language delay (MSEL composite score mean of 50)	PRT + caregiver training	Researchers + caregivers: NR, home	N/A	Expression, social communication: # spontaneous utterances Expression, semantic: MCDI words and sentences Expression, composite: PLS expressive Comprehension, composite: PLS receptive Expression, composite: MSEL expressive Comprehension, composite: MSEL receptive	0.64 0.82 0.17 0.15 0.47 0.15	Strong
Holzinger et al. (2019); Quasi-experimental	7 6	Ex: 42.3 (6) Con: 44.5 (8.2)	0 0	MSEL composite score (Ex: M = 57.7, SD = 13.5; Con: M = 48, SD = 10.6)	ESDM	Therapists: NR, home	N/A	Expression, composite: MSEL expressive Comprehension, composite: MSEL receptive Expression, semantic: A-CDI	0.59 0.46 0.61	Adequate • No random assignment, IOA, procedural fidelity, or generalization/maintenance • Sample size for analyses < 10
Mohammadzaheri et al. (2022); Randomized clinical trial	10 10	Ex: 101 (22.6) Con: 103 (22.9)	0 0	Mean MLU = 2.1	PRT	SLPs: various activities, clinic	N/A	Expression, social communication: # spontaneous question asking Expression, syntactic: MLU	5.1 4.25	Strong
Vivanti et al. (2014); Quasi-experimental	27 30	Ex: 40.3 (9.6) Con: 42 (6.7)	17 11	Mean language age 16–20 months (MSEL)	ESDM + caregiver training	Teachers, various activities, classroom	N/A	Expression, composite: MSEL expressive Comprehension, composite: MSEL receptive	0.58 0.50	Strong
*NDBIs Without AAC Compared Against Structured ABA Interventions (NDBI-Only)*										
Mohammadzaheri et al. (2014); Randomized clinical trial	15 15	PRT: 110.7 (18.7) ABA: 110.5 (18.6)	40 40	Mean MLU = 2.8	PRT vs. structured ABA	Researchers: play (PRT) or DDT (ABA), treatment room at school	N/A	Expression, syntactic: MLU	0.63	Strong
Paul et al. (2013); Randomized clinical trial	12 10	MCT: 42 (9.6) RMIA: 51.6 (14.4)	8 40	< 6 words	MCT vs. RMIA (caregiver training for both)	Researchers: play (MCT) or DDT (RMIA), NR	N/A	Expression, semantic: CSBS spoken word inventory	0.02	Adequate • Unclear data analysis • No IOA or blind raters • Minimal procedural fidelity
*Comparisons of NDBI and AAC Interventions (Comparative Studies)*										
Kasari et al. (2014); SMART	30 31	JASP + EMT: 74.2 (13) + SGD: 77.3 (14.8)	13 21	< 20 different words in 20 min	JASP + EMT vs. JASP + EMT + SGD (caregiver training for both)	Researchers: play, clinic	SGD	Expression, social communication: TSCU Expression, semantic: TDWR Expression, social communication: TCOM	0.62 0.29 0.44	Strong
Schreibman and Stahmer (2014); Randomized clinical trial	19 20	PECS: 28.9 (4.2) PRT: 29.5 (6.9)	16 10	< 10 spoken words	PECS vs. PRT (caregiver education for both)	Researchers: play (PRT) or DTT (PECS), home	PECS	Expression, composite: MSEL expressive	0.41	Strong
Yoder and Stone (2006); Randomized clinical trial	19 17	All: 33.6 (8.4)	14	Minimal speech (M = 0.25 utterances/15 min)	PECS vs. RPMT (caregiver education for both)	Researchers: play (RPMT) or DTT (PECS), clinic	PECS	Expression, social communication: frequency of nonimitative speech Expression, semantic: # different nonimitative words	0.63 0.5	Strong

*N* sample size, *M* mean, *SD* standard deviation, *NDBI* naturalistic developmental behavioral intervention, *AAC* augmentative and alternative communication, *DV* dependent variable (language only), *RCT* randomized control trial, *Ex* experimental group, *Con* control group, *MSEL* Mullen Scales of Early Learning, *JASPER* Joint Attention Symbolic Play Engagement and Regulation, *IJA* initiation of joint attention, *ESDM* Early Start Denver Model, *EA* Early Achievements, *PRT* Pivotal Response Training, *NR* not reported, *MCDI* MacArthur-Bates Communicative Development Inventory, *PLS* Preschool Language Scales, *A-CDI* Communicative Development Inventory (Austrian version), *IOA* interobserver agreement, *MLU* mean length of utterance, *SLP* speech-language pathologists, *ABA* applied behavior analysis, *DTT* discrete trial training, *MCT* Milieu Communication Training, *RMIA* Rapid Motor Imitation Antecedent, *CSBS* Communication and Symbolic Behavior Scales, *SMART* sequential multiple assignment randomized trial, *JASP* + *EMT* Joint Attention Symbolic Play + Enhanced Milieu Teaching, *SGD* speech generating device, *TSCU* total number of spontaneous communicative utterances (includes SGD), *TDWR* total number of different word roots (includes SGD), *TCOM* number of comments (includes SGD), *PECS* Picture Exchange Communication System (not considered an NDBI in the manualized form), *RPMT* Responsive Education and Prelinguistic Milieu Teaching

^1^Effect size differences between experimental and control groups, unless otherwise stated; For studies that reported a different effect size or no effect sizes, Cohen’s *d* was calculated

Participants characteristics were similar across all study types. Participant ages ranged from 23 months to 13 years old, with the bulk of participants in the 2–5-year-old range (390/517 = 75%). NDBIs are often targeted at early intervention and preschool/kindergarten services, which aligns with the high proportion of participants in this age range. All participants across all included studies had significant speech and language impairments. However, the average participant age for NDBI+AAC single-case studies was slightly higher than for other study types (79 months vs. 48–57 months). Participants in NDBI+AAC single-case studies also tended to be described as using less speech than other study types (mostly “minimal speech” or “less than 10 spoken words”—see Table 1). Thus, it is possible that there may have been a slightly more notable gap between participants’ chronological age and speech skills for NDBI+AAC studies than NDBI-Only or Comparative studies.

Considering all included studies, 82% of all participants were male (424/517). Only 52% of papers reported participant racial/ethnic data (8 single-case, 7 group), a trend that spanned across publication date (i.e., several recent papers also omitted racial/ethnic data). Within those studies that included participant race and/or ethnicity, 37% of participants were white, 11% Black, 17% Asian, 9% Hispanic/Latino, and 26% other race/multiracial. Two studies (Mohammadzaheri et al., 2014, 2022) were conducted in Iran, including a total of 50 Iranian participants, contributing to 59% of the total other/multiracial participants.

Dependent variables were included within this review if they measured language expression or comprehension via speech or aided AAC over multiple study timepoints (consistently over time for single-case; pre-post for group designs). Measures that *only* encompassed 1) formative aspects of communication (e.g., joint attention, pointing), 2) social pragmatics (e.g., eye gaze, body language), or 3) unaided AAC (e.g., gesture, facial expression) were excluded. However, composite measures that covered multiple modes of communication were included if they contained aided AAC and/or speech as one communication mode. Variables that represented *only* imitated or verbally/physically prompted communication were excluded. Composite measures that represented both spontaneous and prompted/imitated communication were included if data on spontaneous communication were not reported separately.

### NDBI-Only Studies

Studies were classified as NDBI-Only if they evaluated an intervention that used NDBI procedures but did not include aided AAC (NDBI-Only). Nineteen studies (10 single-case, 9 group) investigated NDBIs without the inclusion of aided AAC, either in comparison to participant baseline performance, a treatment-as-usual control group, or structured ABA intervention. NDBI-Only studies accounted for a total of 360 participants.

#### Study Characteristics

Members of the research team trained specifically for the study protocol served as interventionists for almost two thirds of NDBI-Only studies (12/19 = 63%). Most studies took place during play (12/19 = 63%), with school as the most common study context (9/19 = 47%). Almost all NDBI-Only studies cited manualized interventions as the independent variable (e.g., EMT, JASPER, PRT; 17/19 = 89%). Measures of language expression accounted for most outcome variables (32/37 = 86%), with one study including a standardized composite measure of expression and comprehension (Engelstad et al., 2020). Single-case studies mostly targeted criterion-referenced language outcome measures designed for the study, while group studies had a mixture of standardized and criterion-referenced measures. Studies mostly targeted social communication and requesting variables, though both semantic and syntactic outcomes were represented.

#### Certainty of Evidence

Most NDBI-Only studies received a “strong” rating (15/19 = 79%), with the remaining four representing “adequate” certainty, according to the criteria set by Reichow et al. (2008). This level of certainty was consistent across both single-case (8/10 = 80%) and group design (7/9 = 78%) NDBI-Only studies. However, it is important to point out that some of the methodological issues noted within the “adequate” studies still had the potential to significantly impact the interpretation of study outcomes (e.g., insufficient or rising baseline data, lack of blind coders or insufficient procedural fidelity, lack of generalization or maintenance).

#### Intervention Effectiveness

The average Tau-U effect size on language measures for NDBI-Only single-case studies was 0.74 (large; SE = 0.11, 95% CI [0.52, 0.97]). When narrowing the included data to “strong” certainty of evidence only, single-case studies maintained a large effect size (Tau-U = 0.71, SE = SE 0.13, 95% CI [0.46, 0.96]). Group design NDBI-Only research demonstrated a slightly different pattern to the corresponding single-case results, with an aggregate Cohen’s *d* of 1.95 (large; SE = 1.32, 95% CI [− 0.63, 5.53]) with all articles included. However, one study (Mohammadzaheri et al., 2022) was a significant outlier in terms of effect size (study level effect: *d* = 4.68, SE = 1.11). With this study removed, the average effect size across all group NDBI-Only studies was *d* = 0.61 (SE = 0.63, 95% CI [− 0.63, 1.85]), in the lower end of a medium effect. When limited to “strong” evidence only (and excluding the outlier study), the effect size remained consistent (*d* = 0.62, SE = 0.64, 95% CI [− 0.63, 1.87]).

### NDBI + AAC Studies

Studies were classified as NDBI+AAC if they met Tiede and Walton’s (2019) criteria to qualify as an NDBI and also included instruction in, or introduction of, aided AAC as a part of the intervention procedures. All seven NDBI+AAC studies were exclusively single-case designs, including 21 total participants.

#### Study Characteristics

Research team members trained prior to beginning study procedures constituted just over half of interventionists in NDBI+AAC studies (4/7 = 57%). The majority of studies took place during play activities (4/7 = 57%), and predominantly in the school context (5/7–71%). Only one NDBI+AAC study reported using a manualized NDBI (Olive et al., 2007—EMT). Study outcome variables overwhelmingly represented expressive language targets (10/11 = 91%), with only one study including a measure of language comprehension (Drager et al., 2006). All studies targeted criterion-referenced language outcome measures designed for the study, likely in large part due to the frequent testing paradigm of single-case research. As with NDBI-Only studies, social communication and requesting were the most common target outcomes, though both semantic and syntactic dependent variables were represented.

All NDBI+AAC studies provided relatively detailed information regarding the characteristics of the aided AAC systems. Three studies used high-tech dynamic display speech generating devices (3/7 = 43%; SGDs), two included mid-tech systems with physical buttons and pre-recorded speech output, and two studies incorporated low-tech symbols. Most systems used line drawings to represent vocabulary (5/7 = 71%). One study took color photographs of book concepts to use as symbol representations (Finke et al., 2017), one participant within a study used a keyboard on a tablet to type his responses (Carnett et al., 2020), and one study did not specify the symbol type (Olive et al., 2007). All but one AAC system was comprised of multiple vocabulary symbols in a grid display (Johnston et al., 2003).

### Certainty of Evidence

NDBI+AAC interventions demonstrated more variable certainty of evidence, with just over half receiving a “strong” rating (4/7 = 57%) and the remaining three exhibiting “adequate” certainty, based on the criteria set by Reichow et al. (2008). See Tables 1 and  2 for information relating to ratings of “adequate” for specific studies.

### Intervention Effectiveness

The average Tau-U effect size on language measures for NDBI + AAC studies (all single-case) was 0.85 (very large; SE = 0.15, 95% CI [0.56, 1.15]). When only studies rated to have “strong” certainty of evidence were included, NDBI + AAC studies had a slightly lower average effect size of 0.71 (SE = 0.23, 95% CI [0.36, 1.26]), within the large range.

### Comparative Studies

Three studies identified via the systematic search procedures directly compared NDBIs without AAC to either: 1) that same NDBI with AAC added (Kasari et al., 2014) or 2) a manualized AAC intervention that did not qualify as an NDBI (the Picture Exchange Communication System—PECS; Schreibman & Stahmer, 2014; Yoder & Stone, 2006). All three studies were group designs, encompassing a total of 136 participants.

#### Study Characteristics

Similar to the majority of NDBI-Only and NDBI + AAC studies, intervention activities in all Comparative studies were conducted by trained researchers within a play-based context. Two studies took place in a clinical setting, and one within the home, and all three utilized manualized NDBI procedures. All outcome measures targeted expressive language (6/6 = 100%), though there was variety across studies in the domain targeted (e.g., social communication, requesting) and the type of measure (e.g., standardized assessment, criterion referenced measure).

Descriptions of the aided AAC systems included in the three Comparative studies were extremely limited. Two studies simply reported using PECS, and the third a speech-generating device (SGD). Information on specific vocabulary items, layout, organization, or symbol representation was not included. This contrasts with the relatively detailed descriptions of the AAC systems used in NDBI + AAC studies.

#### Certainty of Evidence

All Comparative studies received a certainty of evidence rating of “strong,” according to the criteria set by Reichow et al. (2008), indicating that any differences in the dependent measure(s) (in this case, language variables) were likely attributable to the independent variable (i.e., the different interventions tested). However, the lack of description of included AAC systems within all three studies limits the replicability of the procedures.

#### Intervention Effectiveness

The three Comparative studies allowed for calculation of the relative effectiveness of (a) NDBI-Only vs. NDBI + AAC (Kasari et al., 2014) and (b) NDBI-Only vs. PECS (a non-NDBI, ABA-based AAC intervention) on dependent language measures. The Cohen’s *d* effect size for adding aided AAC into NDBI procedures on reported language measures in Kasari et al. (2014) ranged from 0.29 to 0.62, indicated a small to medium effect on language growth by simply including aided AAC within the preexisting NDBI methodology (see Table 3). This suggests that incorporating aided AAC into the targeted NDBI noticeably bolstered children’s language growth even further, above and beyond the effect of the NDBI procedures alone.

**Table 3:** Effect sizes for intervention types and intervention comparisons

Intervention design (or study)	Effect size: all studies	Level of effect	Effect size: strong only	Level of effect
*NDBI-only*				
Single-case	0.74	Large	0.71	Large
Group	1.95*	Large	2.03*	Large
*NDBI + AAC*				
Single-case	0.85	Very large	0.81	Very large
*Comparative studies*^*1*^				
Kasari et al. (2014)	0.29–0.62	Small to medium		
Schreibman and Stahmer (2014)	0.41	Small		
Yoder and Stone (2006)	0.5–0.63	Small to medium		

Effect sizes for single-case designs are Tau U (small = 0.2; medium = 0.21–0.6; large = 0.61–0.8; very large =  > 0.8; Vannest & Ninci, 2015); Effect sizes for group designs are Cohen’s *d* (conventions: small = 0.2; medium = 0.5; large = 0.8; Cohen, 1992); *SD* standard deviation, *NDBI* naturalistic developmental behavioral intervention, *AAC* augmentative and alternative communication

^*^Cohen’s *d* with single outlier study removed = 0.61 (all), 0.62 (strong only)

^1^Includes only studies that directly compared an NDBI-Only to and NDBI + AAC or non-NDBI AAC intervention (Kasari et al., 2014; Schreibman & Stahmer, 2014; Yoder & Stone, 2006)

The Cohen’s *d* for NDBIs vs. PECS (Schreibman & Stahmer, 2014; Yoder & Stone, 2006) ranged from 0.41–0.63, indicating a small to medium effect of PECS on language growth when compared to the NDBIs. This is especially noteworthy considering that there is evidence within the research included in this review that NDBIs may be more effective at positively impacting language development than ABA-based interventions that do not include aided AAC (Mohammadzaheri et al., 2014; Cohen’s *d* of PRT vs. ABA = 0.66, a medium effect). These results suggest that access to AAC within language interventions may have a larger positive impact than NDBI procedures alone on the language development of young children on the autism spectrum.

### Comparative Effectiveness of NDBI-Only and NDBI + AAC Interventions

Both NDBI-Only and NDBI + AAC studies showed strong positive effects on participants’ language. However, the average effect size for single-case NDBI + AAC studies (Tau-U = 0.85) was larger than that for single-case NDBI-Only studies (Tau-U = 0.74). The average effect size for group NDBI-Only studies was in the large range for Cohen’s *d* (1.95; SE = 1.32, 95% CI [− 0.63, 5.53]), though this value appeared to be influenced by a single outlier (Mohammadzaheri et al., 2022). With this study removed, the NDBI-Only group average effect size was in the medium range (Cohen’s *d* = 0.61, SE = 0.63, 95% CI [− 0.63, 1.85]), and maintained a similar effect size when only studies rated as strong were included (Cohen’s *d* = 0.62, SE 0.64, 95% CI [− 0.63, 1.87]). However, it is important to note that Tau-U and Cohen’s *d* are not directly comparable.

Additionally, the results of the included Comparative studies demonstrated a small to medium effect of aided AAC intervention on language outcomes beyond that of traditional NDBIs that did not include aided AAC (Cohen’s d range: 0.29–0.63). It is notable that these results follow the same pattern as the average effect sizes of NDBI-Only as compared to NDBI + AAC studies. Taken together, these results suggest that the inclusion of aided AAC within NDBI methods may increase the positive impact of NDBIs on the language development of children on the autism spectrum.

However, results of the aided AAC moderator analysis that included all single-case studies (NDBI-Only and NDBI + AAC) were not statistically significant (Tau-U = 0.11, SE = 0.19, p = 0.59, 95% CI [− 0.26, 0.48]). This outcome of the moderator analysis suggests that while the average effect size of included single-case NDBI + AAC studies may be larger than that of the single-case NDBI-Only studies, the unique contribution of aided AAC on this discrepancy is not a strong enough direct moderator on language outcomes to reach statistical significance in this sample.

It is important to note that comparisons across these different studies and study types are exploratory in nature. Variation in participant characteristics, study procedures, dependent language measures, and AAC systems limits the direct interpretability and comparability of the aggregate effect size data of NDBI-Only and NDBI + AAC studies. On average, participants in NDBI + AAC studies were slightly older, and were generally reported to have less functional speech than participants in many (though not all) of the NDBI-Only studies. NDBI-Only studies were also much more likely to report using pre-established manualized NDBIs (e.g., PRT, EMT), whereas this was the case for only one NDBI + AAC study (Olive et al., 2007). By contrast, both NDBI-Only and NDBI + AAC studies tended to take place during play, at school, and with research personnel serving as the interventionist. Almost all variables across all studies represented expressive language measures. However, it is unknown whether any critical characteristics may have varied systematically between NDBI-Only and NDBI + AAC studies that could have contributed to the observed differences in aggregate effect sizes between study types.

### Risk of Publication Bias

**Fig. 2:**
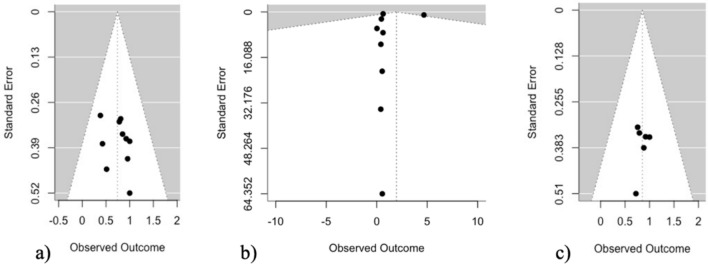
Funnel plots representing risk of publication bias for **a** NDBI-Only single-case study, **b** NDBI-Only group studies, and **c** NDBI + AAC single-case studies

Funnel plots were created using R (R Core Team, 2023) to assess risk of publication bias for NDBI + AAC single-case studies, NDBI-Only single-case studies, and NDBI-Only group studies (Fig. 2). For the funnel plots for NDBI-Only single-case studies and NDBI + AAC single-case studies (Fig. 2a and c), estimate effect sizes for individual studies (represented by dots on the graph) are relatively equally distributed around the vertical line that represents the estimated overall effect across included studies in that group (e.g., NDBI-Only group). This indicates minimal risk of bias, suggesting the studies in these categories are less likely to be biased towards publication of studies with positive results. In the funnel plot for NDBI-Only group studies (Fig. 2b), individual study effect sizes are clustered near the average effect line, though all but one effect size falls to the left of (i.e., below) the average effect line. One study with a higher effect size appears to be potentially inflating the average effect. However, this may suggest that there is a publication bias toward studies with more modest effects. Note that several individual measures in some NDBI-Only group studies (b) reflected number of words produced from a corpus of 300 + (e.g., on the MCDI), with very high variance across study participants. As a result, the full breadth of the x-axis scale 95% confidence interval pyramid was truncated in the presented plot, to allow for better visualization of the distribution of included studies around the average effect size. All studies are visible within the included portion of the larger funnel plot.

## Discussion

Both traditional NDBIs *without* AAC (NDBI-Only) and NDBIs that included AAC (NDBI + AAC) were effective at promoting language growth in children on the autism spectrum, in line with results from previous meta-analyses (Crank et al., 2021; Sandbank et al., 2020; Tiede & Walton, 2019). These results provide further evidence that interventions blending the critical elements of structured, behavior-based interventions into developmentally appropriate and naturalistic environments can have a powerful impact on the language development of young children on the autism spectrum.

However, both direct (Comparative studies) and indirect (average Tau-U values from NDBI-Only and NDBI + AAC research) comparative evidence from this review suggests the greater power on language and communication of including aided AAC within NDBI procedures. While this relationship was not strong enough for meta-analytic moderator analysis to show a significant moderator effect of aided AAC on language outcomes, the trend was consistent across all comparison methods. Without access to effective communication supports, children with minimal speech cannot participate fully within the rich language learning environment of an NDBI. As a result, these children with the greatest communication needs make the fewest gains (Kasari et al., 2014). They cannot readily practice vocabulary modeled by communication partners, and thus miss out on critical feedback on their own utterances. Without an effective means of expression, the reciprocal interaction and language play characteristic of early language learning is substantially limited for these children, putting them at significant risk in terms of developing the diverse initial vocabulary needed to support later word combinations, negatively impacting long-term language trajectories (Tek et al., 2014).

Importantly, studies that both did and did not include aided AAC demonstrated marked similarities in terms of their intervention procedures, participant language skills, and language measures, indicating that NDBIs may be able to incorporate AAC systems into their procedures with minimal adjustments, as demonstrated by Kasari et al. (2014). Evaluation of average effect size data across intervention types within this review, as well as direct comparative studies, suggests that when AAC systems *are* included within NDBIs, the language skills of children on the autism spectrum may improve to a greater extent than with NDBI procedures on their own. In fact, the only current comparative study directly investigating the added effect of incorporating aided AAC into a manualized NDBI (Kasari et al., 2014) clearly demonstrates that this type of integration is both feasible and highly effective at significantly bolstering language and communication growth, above and beyond the impact of NDBI procedures alone.

However, the same considerations for developmentally appropriate intervention and transparent and replicable procedures must extend from the manualized NDBI protocols themselves to the integrated AAC systems. The features of an AAC system can impact the communication success of children on the autism spectrum (Ganz et al., 2014), and the match between an AAC system’s features to the strengths and needs of each individual child is critical (Light & Drager, 2007). Of the three Comparative studies that followed manualized NDBI procedures and included aided AAC, little description of the AAC systems was available. Kasari et al. (2014) described the AAC system(s) only as “SGDs,” referencing two types of high-tech devices (iPads and DynaVox devices), and that vocabulary was relevant to the toys/activities included in intervention. Schreibman and Stahmer (2014) and Yoder and Stone (2006) simply stated that participants used “PECS.” No information was provided regarding how vocabulary was represented (e.g., pictures, line drawings), organized (e.g., grid displays, visual scene display, on one page or multiple, the number of symbols per page), or the parts of speech included (e.g., nouns, verbs, descriptors). All of these factors can contribute to the success of AAC intervention. Equal consideration of both the features of aided AAC systems—and the goodness of fit to children’s developmental level—as well as the specific NDBI procedures is essential.

### Implications for Research and Practice

The results of this review demonstrate that the children participating in NDBI research may benefit from access to aided AAC, and that when AAC is incorporated into intervention, language outcomes are increased. Similarly, cumulative evidence indicates that AAC intervention does not negatively impact speech development for children on the autism spectrum and may even facilitate spoken language growth (see Alzrayer et al., 2021, included in this review; Schlosser & Wendt, 2008). Additionally, the intervention, participant, and outcome variable characteristics were similar across studies that included AAC and those that did not, suggesting that AAC systems may be incorporated into preexisting NDBI protocols with minimal alterations (see Kasari et al., 2014). These considerations apply equally to future research and current clinical practice. Given this evidence, considering aided AAC as a valuable and compatible component within NDBIs appears to require minimal adaptation, with maximal potential benefits on child language.

However, it is important than when AAC systems are included in intervention, this is done so thoughtfully and with a developmental perspective, sensitive to the skills and needs of each child. Additionally, when incorporated into research, it is essential that AAC systems are described sufficiently, to help elucidate what works, for whom, and under what conditions. Critically considering and fully describing the features of AAC systems can create the most potential for positive impacts on language and offer concrete guidance for future research and clinical practice.

Given the results of this systematic review, both researchers and clinicians are urged to consider integrating aided AAC into NDBI procedures as a powerful intervention approach for supporting the language development of young children on the autism spectrum with minimal speech (see Table 4 for examples of integrating aided AAC into NDBI strategies). Prior research evidence also demonstrates that including aided AAC within an NDBI from the outset of intervention is significantly more effective than considering AAC as a backup measure to be added after the fact, if traditional NDBI procedures alone are minimally effective (Kasari et al., 2014). In addition, typically developing toddlers as young as 10 months of age are able to use developmentally appropriate AAC technology expressively—and even participate in programming in new vocabulary (Holyfield et al., 2017)—suggesting that introduction of even high tech aided AAC early in development is effective and appropriate, when those AAC systems are designed to support beginning communicators (e.g., visual scene displays with just-in-time programming; Holyfield et al., 2017). For any children with minimal speech on the autism spectrum participating in an NDBI, it is crucial that the included AAC systems are thoughtfully designed to meet their specific strengths and needs.

**Table 4:** Integrating AAC into NDBI strategies (examples)

NDBI strategy	Description	Integration of AAC
Setting up an interactive context	Remove distractors in the environment; ensure activity materials are accessible to child and/or strategically out of reach	Remove distractors in the environment; ensure activity materials are accessible to child and/or strategically out of reach; *ensure AAC is accessible to child*
Following the child’s lead while supporting balanced turns	Respond to child’s interests during the activity; take turns during the activity	Respond to child’s interests during the activity, *including providing aided AAC modeling*; take turns during the activity, *including providing aided AAC modeling*
Least-to-most prompting	Provide increasingly more supportive prompts to encourage the child to engage (e.g., expectant delay → gestural prompt → vocal prompt → model, as required)	Provide least-to-most prompting to encourage child to engage, *including prompts to use the AAC system for expression*
Modeling language	Use spoken language relevant to the activity; draw the child’s attention to relevant actions/objects/people/etc	Provide spoken language relevant to the activity, *also using the AAC system to model language*; draw the child’s attention to relevant people, actions, and objects, etc., *pairing them to the relevant AAC vocabulary *via* aided AAC modeling*
Providing event casts	Describe the child’s actions in the moment using short spoken utterances (e.g., “You’re driving the car!”)	Describe the child’s actions using spoken utterances *and aided AAC modeling* (e.g., “You’re driving the car!” [AAC: DRIVE + CAR])
Expanding on child communication	Restate child utterances in a slightly longer spoken phrase (e.g., Child: “Car!”; Adult: “Drive the car!”)	Expand child utterances using slightly longer spoken phrases *and aided AAC modeling* (e.g., Child: “Car!”; Adult: “Drive the car!” [AAC: DRIVE + CAR])
Natural and child-contingent reinforcement	Reinforce the child in ways that align with the interaction (e.g., the child asks for the car and is given the car), and reinforce (e.g., getting the car) appropriate child behavior (e.g., requesting the car in some way)	Reinforce the child in ways that align with the interaction (e.g., the child asks for the car and is given the car), and reinforce (e.g., getting the car) appropriate child behavior (e.g., requesting the car in some way), *including aided AAC modeling* (e.g., AAC: WANT + CAR)

### Limitations and Future Directions

Although this review offers a novel contribution in bridging the research evidence on NDBIs and AAC intervention for children on the autism spectrum, there are several limitations. It is possible that studies targeting relevant language measures that would meet the criteria of an NDBI were not captured in the search procedures. This may be especially true for AAC interventions, in which NDBI-based terminology is less common. This exclusion bias was mitigated in part by consulting previous papers and systematic reviews capturing naturalistic AAC intervention research more broadly (e.g., Gevarter & Zamora, 2018). Additionally, only peer-reviewed articles were included, minimizing the contributions of dissertation and thesis research, and potentially resulting in conclusions influenced by publication bias. However, funnel plots of included studies suggest publication bias may be minimal. Caregiver—and peer-mediated interventions were also not included in this review, though they are not uncommon in NDBI or AAC research. The review was limited to professional interventionists (e.g., researchers, teachers, teaching assistants, clinical therapists) to allow for better comparison across intervention types. Some evidence suggests that caregiver-mediated NDBIs may not be as effective on language development as those completed by researchers and education professionals (Sandbank et al., 2020), though peer-mediated AAC interventions may be especially successful for children with complex communication needs generally (Gevarter & Zamora, 2018). Future research should investigate how caregiver—and peer-mediated NDBIs interact with AAC to support the language development of children on the autism spectrum.

Only one AAC study reported using a manualized NDBI (EMT – Olive et al., 2007). The small representation of AAC research within this NDBI review highlights the substantial disconnect between these two areas of study, both of which are focused on promoting the language development of children on the autism spectrum. The results of this study provide further evidence that adding aided AAC systems into NDBI methods may increase the language outcomes of participating children on the autism spectrum, underscoring the critical importance of bridging the gap between these two areas of research and practice. Additionally, the methodological rigor and reach of AAC research may be bolstered by explicitly incorporating manualized NDBI procedures or partnering with NDBI researchers, strengthening the evidence base for AAC as an essential component in supporting the language development of children on the autism spectrum with minimal speech.

Additionally, while the majority of studies in this review received a “strong” certainty of evidence rating based on the criteria set by Reichow et al. (2008), previous meta-analytic research targeting similar topics has indicated a greater need for improvements in terms of methodological rigor (e.g., Crank et al., 2021; Sandbank et al., 2020). These differences may stem from several factors. First, importantly, though several of the included studies were rated as “adequate” based on the criteria from Reichow et al. (2008), some of the noted design flaws could have theoretically had a significant effect on the study outcomes. Additionally, these guidelines from Reichow et al. (2008) focus on autism intervention research specifically, but include criteria for assessing both single-case and group design research. This was highly relevant to the current study aims, though related meta-analyses that focused on group-based research only may have had more stringent criteria, but that were applicable to group designs only. Lastly, the current study excluded caregiver—and peer-mediated interventions. While these types of studies can be undertaken with high degrees of methodological rigor, it is possible the inclusion of these studies in related meta-analyses could have decreased the overall quality of the evidence.

The results of this review are based overwhelmingly on expressive language measures, with only one study including a language comprehension target beyond a standardized assessment (Drager et al., 2006). Challenges in both language comprehension and expression are common for children on the autism spectrum and evidence suggests that supporting language comprehension can promote both expressive language growth and literacy development (Muller & Brady, 2016). A greater focus on directly targeting and assessing language comprehension is needed in both intervention research and clinical practice (Muller & Brady, 2016). This review provides consistent evidence that NDBIs can have a significant positive effect on language comprehension. However, these results are limited in scope and based mostly on standardized assessments (Dawson et al., 2010; Drager et al., 2006; Holzinger et al., 2019; Vivanti et al., 2014). Future research would benefit from greater emphasis on language comprehension targets, both to expand the knowledge base on the effectiveness of NDBIs on comprehension skills, as well as to provide evidence and guidance for clinical practice.

The features of an AAC system itself can impact the communication success of children on the autism spectrum (Ganz et al., 2014), as can intrinsic child factors and extrinsic intervention characteristics (Sievers et al., 2018). All studies in this review provided general descriptions of relevant child and intervention factors. Additionally, single-case studies that included aided AAC offered at least some details regarding the system(s) used. However, among group Comparative studies, information on AAC systems was negligible.

The clinical significance of the measurable increase in child language growth accompanying the inclusion of AAC is limited by insufficient description of the included systems. These generalized conclusions regarding AAC may lead clinicians to assume that *any* AAC system can be added to language intervention for *any* child on the autism spectrum with minimal speech, with equally positive results. However, the goodness-of-fit of an AAC system’s features to the strengths and needs of each individual child is essential (Light & Drager, 2007). Nonetheless, the aggregate effect size of these three Comparative studies still represented a small to medium effect of AAC on language (Cohen’s *d* range: 0.29–0.63), over and above NDBIs alone, even without consideration of specific AAC features and goodness-of-fit for participants. This suggests that the impact of integrating well-designed AAC systems into NDBI instruction for children on the autism spectrum may be even greater. Future research exploring the comparative effectiveness of NDBIs with and without aided AAC is needed that includes careful documentation of AAC system variables to better understand which AAC interventions work best for whom under what conditions.

ASD research continues to be focused on participants who are young children, white, and male (Watkins et al., 2014; West et al., 2016). The included studies demonstrated similar trends within NDBI and AAC research, with 82% male participants and only slightly more than half of studies even including racial and ethnic information in participant descriptions. Whether interventions are equally effective—or how they can be adapted—for underrepresented racial, ethnic, linguistic, gender, and age groups is a question that can only be answered if these groups are intentionally included within research studies that make a point to report on these relevant identity characteristics. Given the intersection of race, ethnicity, gender, and disability (Blanchett et al, 2009), it is crucial that the ASD research literature begin to explore the unique strengths and needs of these underrepresented groups.

While overall presenting a degree of consistency across study types (NDBI-Only, NDBI + AAC, Comparative), the research designs, intervention procedures, participant characteristics, and language measures of the included studies varied. For example, participants in NDBI + AAC studies were slightly older, and were generally reported to have less functional speech than participants in NDBI-Only and Comparative studies. NDBI-Only and Comparative studies were also much more likely to report using pre-established manualized NDBIs (e.g., PRT, EMT) than NDBI + AAC studies. This variability restricts the interpretability or comparability of average effect size values. Additionally, it is possible that some critical characteristic(s) may have varied systematically between NDBI-Only and NDBI + AAC studies, potentially contributing to the observed differences in average effect sizes between study types, unrelated to the inclusion of aided AAC. Additional randomized clinical comparisons are needed to draw meaningful conclusions regarding the relative effectiveness of NDBI interventions with and without AAC as a component on the language skills of children on the autism spectrum (e.g., Kasari et al., 2014).

## Conclusion

The results of this review indicated that while NDBI procedures are effective for promoting the language development of young children on the autism spectrum, when AAC is incorporated into these NDBIs, it may lead to a measurable additional increase in language growth—above and beyond that of NDBIs alone.
